# Chemokines in Pregnancy

**DOI:** 10.3390/biom15121645

**Published:** 2025-11-22

**Authors:** Julia A. Shevchenko, Alina A. Gizbrekht, Sergey V. Sennikov

**Affiliations:** Laboratory of Molecular Immunology, Federal State Budgetary Scientific Institution “Research Institute of Fundamental and Clinical Immunology” (RIFCI), 630099 Novosibirsk, Russia; shevchenkoja2023@yandex.ru (J.A.S.); gizbrekht.aa17@gmail.com (A.A.G.)

**Keywords:** pregnancy, chemokine, erythroid cell, preeclampsia, feto-maternal tolerance

## Abstract

Cell migration capacity represents an essential function of the immune system. Pregnancy involves numerous morphogenetic events, angiogenesis, the establishment of intercellular connections, and complex interactions between maternal and fetal immune systems—all requiring precisely coordinated and regulated migratory processes. Chemokines serve as master regulators of cellular migration and communication during pregnancy, with functions extending far beyond classical immunological roles. Physiological chemokine levels maintain feto-maternal tolerance through precise spatiotemporal regulation, while their dysregulation leads to catastrophic pregnancy complications such as preeclampsia and preterm birth. The chemokine system exhibits remarkable complexity through functional redundancy and promiscuity of receptors and ligands; alternative splicing generating protein diversity; decoy receptors enabling the fine-tuning of chemokine concentrations; and heterocomplex formation creating novel biological functions. Chemokines show significant promise as diagnostic and prognostic biomarkers, while specific receptor–ligand pairs represent therapeutic targets for managing pathological and life-threatening conditions during pregnancy. Thus, the chemokine system constitutes both a fundamental biological mechanism supporting pregnancy and a promising translational target for addressing complex clinical challenges in obstetric complications. To fully harness the potential of this system, it is essential to understand both its evolutionarily conserved core functions and its gestational stage-specific adaptations.

## 1. Introduction

Cell migration represents a critical function of the immune system, enabling essential mobility for host defense against microbial invasion or malignant cells. Migration processes are active from early mammalian development when hematopoietic progenitor cells migrate from the yolk sac to colonize tissues. Later stages involve waves of progenitor and monocyte migration from bone marrow to secondary lymphoid organs and peripheral tissues. In adulthood, cell migration allows immune cells to patrol their environment and circulate between peripheral tissues and lymphoid organs. Thus, immune cell migration is fundamental for establishing an effective host defense system [1].

Signal generation initiates directed migration, where signals may be transient (e.g., diffusible chemical cues inducing short-term migration) or sustained (e.g., environmental modifications guiding long-term cell movement). Chemotaxis is mediated by soluble factors through diffusion. Under uniform signal distribution, cells undergo chemokinesis—random migration with increased speed and/or turning frequency compared to unstimulated cells—whereas gradient-dependent signals induce directed migration [2].

Pregnancy involves extensive morphogenesis during organ and tissue formation, intercellular communication, and cell exchange between maternal and fetal organisms. Consequently, coordinating these processes requires precise, multicomponent regulation.

## 2. The General Chemokine System

Chemokines constitute the largest cytokine family, characterized by chemotactic activity essential for both physiological and pathological processes. Originally identified as mediators recruiting immune cells to inflammatory sites, subsequent research has demonstrated that chemokine signaling orchestrates neuronal migration, neural crest cell movement, and germ cell trafficking during embryonic development while regulating vascular patterning, tissue remodeling, and cancer metastasis [3]. Dysregulation of this system increases susceptibility to various pathologies including inflammatory disorders, infections, and malignancies [4], with the discovery of chemokines as potent chemoattractants representing a pivotal milestone in immunology [5].

The human chemokine system comprises over 48 members (53 in mice), classified into four structural subfamilies: CC, CXC, XC, and CX3C. CXC chemokines exhibit functional dichotomy based on the presence of the ELR (Glu-Leu-Arg) motif—ELR-positive members (e.g., CXCL8) preferentially attract neutrophils, while ELR-negative variants (e.g., CXCL13) typically recruit lymphocytes [4]. Chemokine receptors belong to the rhodopsin-like GPCR superfamily, with 19 signaling receptors currently identified: 6 CXCR (CXCR1-6), 10 CCR (CCR1-10), CX3CR1, and XCR1 [6]. The system also includes four atypical receptors (ACKR1-4) that utilize β-arrestins rather than G-proteins, characterized by modifications or absence of the conserved DRYLAIV motif and consequent decoupling from classical signaling pathways. While predominantly expressed on endothelial and stromal cells, certain leukocyte subsets also express these regulatory receptors [7].

Chemokines can interact with other proteins and even nucleic acids to form heterocomplexes, potentially resulting in novel biological functions [8]. The chemokine system, as one of the most ancient components of the immune system [9], has acquired a highly diverse set of interconnected functions characterized by redundancy and nonspecficity: chemokine receptors bind multiple ligands, while a single ligand can interact with different receptors. Ligands interacting with the same receptor, particularly when expressed on different cells, may exhibit significant variations in receptor affinity, ability to activate downstream transcriptional factor cascades, susceptibility to modifications, or organ/extracellular matrix presence specificity. Alternative splicing represents another mechanism generating chemokine and receptor multifunctionality, serving as a source of protein diversity in the immune system that alters structure, stability, and turnover rates of transcripts and their encoded proteins. This mechanism controls binding properties, enzymatic activity, and stability of numerous protein isoforms originating from a single gene [10]. A third mechanism for controlling and regulating cytokine activity involves the presence of surface decoy chemokine receptors that can bind chemokines without activating downstream signaling pathways. Effective inflammatory cell recruitment requires low chemokine levels in the bloodstream and high chemokine concentrations in target tissues [11].

Beyond their immunological functions, chemokines play critical roles throughout human reproduction, participating in menstrual cycle regulation, ovulation, embryo implantation, cervical maturation, parturition, and endometriosis pathogenesis [12]. Their involvement spans physiological processes requiring precise cellular navigation (e.g., trophoblast invasion) and pathological conditions featuring dysregulated cell migration (e.g., endometriotic lesion formation). The chemokine system’s evolutionary conservation across these diverse functions underscores its fundamental role in reproductive biology, where it mediates complex cellular interactions through graded signals, receptor-specific activation, and microenvironmental modulation. This molecular framework enables coordinated cellular trafficking essential for successful reproduction while maintaining capacity for rapid adaptation to physiological demands and pathological threats.

## 3. Chemokines in the Decidualization Process

Pregnancy represents a complex biological process comprising distinct stages, including decidualization, implantation, and placentation [13], each requiring precise immune coordination to prevent rejection of the semi-allogeneic fetus. Endometrial decidualization constitutes a crucial process in human endometrium, involving profound morphological and functional differentiation of endometrial stromal cells. Impairment of this process leads to various complications throughout gestation [14,15]. Transcriptomic and secretome analyses in recent decades have revealed dynamic changes in signaling molecules and their intermediates, transcription factors, hormones, growth factors, cytokines, chemokines, adhesion molecules, ligands and receptors, cytoskeletal organization, extracellular matrix (ECM) composition, ion and water transporters, cell cycle regulators, angiogenic factors, and neuropeptides during decidualization. The decidua plays a vital role in establishing immune tolerance toward the semiallogeneic fetoplacental unit and protects the conceptus from maternal immune responses [16].

The decidua, as the maternal component of the placenta in direct contact with fetal trophoblast, governs immune interactions at the maternal–fetal interface [17]. During decidualization, increased expression of process-associated genes such as CXCL12 and CXCL14 is observed. Investigation of chemokines recruiting activated T-cells revealed that decidual stromal cells do not express CXCL9 and maintain CCL5 expression during decidualization, but significantly downregulate CXCL10 and CXCL11 expression. This pattern reflects the functional specialization of these chemokines: CXCL9, CXCL10, and CXCL11 recruit activated lymphocytes with abortifacient potential, while CCL5 facilitates physiological trophoblast invasion into the decidua and recruitment of regulatory T-cells [18].

The decidualization process reduces the cellular capacity to produce T-cell chemotactic factors under inflammatory conditions. The inability to produce CXCR3 ligands and CCL5 is not explained by reduced NF-κB or STAT1 activation, indicating that chemokine expression defects in decidual stromal cells are gene-specific and independent of inflammatory signaling per se [19].

The key chemokine CXCL12, through interaction with its receptor CXCR4, modulates endometrial receptivity, decidualization, vascular remodeling, uterine natural killer cell recruitment, and placental and embryonic development [20]. CXCL12 and CXCR4 activate multiple signaling cascades including the PI3K/AKT, MAPK, PKC, and JAK/STAT pathways, mediating cell proliferation, apoptosis, and autophagy. Autophagy of cellular organelles promotes bioenergy recycling, providing cells with sufficient energy for intensive proliferation and differentiation. In human decidual stem cells during the secretory phase, an inverse correlation exists between autophagy levels and CXCL12 concentration, while inhibition of CXCL12/CXCR4 signaling enhances autophagy at the utero-placental interface, thereby influencing angiogenesis [21].

The chemokine CXCL16 is expressed in normal endometrium and at the maternal–fetal interface, with higher transcript and translation levels observed in decidual tissues and chorionic villi from normal pregnancies compared to endometrium. CXCL16 promotes in vitro decidualization of embryonic stem cells via the PI3K-PDK1-AKT-Cyclin D1 signaling pathway. Notably, reduced expression of both CXCL16 and CXCR6 was observed in decidua and chorionic villi from patients with spontaneous abortion [22].

**Figure 1:**
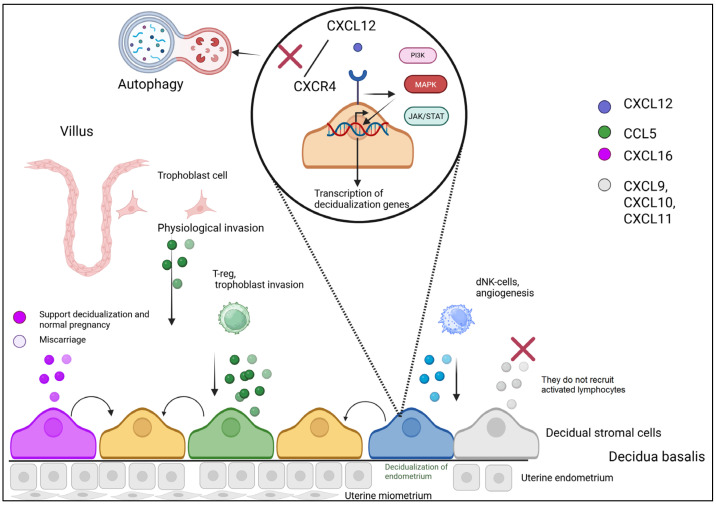
Chemokine-mediated establishment of the decidual immune microenvironment.

The decidua contains substantial leukocyte populations comprising approximately 30–40% of decidual cells. Decidual NK cells (dNK) represent 50–90% of total decidual lymphoid cells during the first trimester [23]. Decidual NK cells migrate in response to CXCL9, CXCL10, and CXCL12 via CXCR3 and CXCR4 receptors. Furthermore, dNK cells migrate through stromal cells in response to CXCL10 and CXCL12 [24] (Figure 1).

During decidualization, stromal cells secrete CXCL12 and CCL5 to recruit immune-tolerant decidual NK cells (dNK) and regulatory T cells (Treg), respectively. Concurrently, the selective suppression of pro-inflammatory chemokines (CXCL9–11) prevents the influx of activated lymphocytes. Trophoblast cells invade maternal tissues. Ligand–receptor interaction between CXCL12 and CXCR4 activates intracellular signaling cascades (PI3K/AKT, MAPK, and JAK/STAT), which regulate key decidualization processes: gene transcription, proliferation, apoptosis, and autophagy. These pathways directly control the secretion of immunomodulatory chemokines (CXCL12 and CCL5) and the suppression of proinflammatory chemokine transcription (CXCL9–11). This coordinated action, driven by intracellular signaling pathways, creates a privileged microenvironment conducive to trophoblast invasion and pregnancy maintenance.

## 4. Chemokine-Mediated Mechanisms in Uterine Spiral Artery Remodeling

During the first trimester of pregnancy, uterine spiral arteries undergo extensive structural transformation, evolving from small-diameter vessels characterized by low blood flow and high resistance into large-diameter conduits with high flow capacity and low resistance. This physiological adaptation confers the crucial advantage of vasoconstrictor insensitivity, thereby ensuring adequate fetal perfusion even during maternal hypotension [25]. The remodeling process is driven by extravillous trophoblast cells, which migrate into vascular smooth muscle layers, endothelial cells, and elastic membranes, ultimately replacing these components to fundamentally reconstruct the uterine spiral arteries [26].

Adequate spiral artery remodeling represents an essential prerequisite for establishing proper blood supply at the maternal–fetal interface. A cornerstone of this process involves the comprehensive replacement of spiral artery endothelial cells by endovascular extravillous trophoblasts. Chemokines serve as pivotal coordinators of both immune and remodeling processes during trophoblast invasion and vascular transformation. While the physical replacement of vascular cells by trophoblasts is well-described, the pivotal coordinating role of chemokines in guiding both immune cells and trophoblasts during this transformation has only recently been fully appreciated.

CXCL12, delivered via extracellular vesicles, induces the migration of decidual natural killer (dNK) cells that play critical roles in vascular remodeling. Trophoblasts secrete a broad spectrum of chemokines—including CCL2, CCL7, CCL20, CXCL1, CXCL2, CXCL5, CXCL6, CXCL8, and CXCL10—that recruit monocytes, macrophages, and other leukocytes to the implantation and remodeling site. Endovascular trophoblasts specifically secrete chemokines that promote M2 polarization: RANTES (CCL5), MIG (CXCL9), and MIP-3α (CCL20). The resulting M2 macrophages perform anti-inflammatory functions, phagocytose cellular debris, and facilitate vascular smooth muscle cell dedifferentiation. Chemokine gradients direct immune cells (including dNK cells and macrophages) to specific locations where they interact with trophoblasts and smooth muscle cells. The secretion of chemokines such as RANTES helps establish a local microenvironment supporting immune tolerance toward the semiallogeneic fetus [27].

Research utilizing 3D vascular spheroids has demonstrated that trophoblast-derived factors significantly alter the expression of 101 genes, with chemokine genes CXCL10 and CCL20 showing more than 2-fold changes. The most significantly altered genes were associated with vascular development. Co-expression of CXCL10 and α-smooth muscle actin in decidual cells indicates that CXCL10’s primary function in vascular remodeling involves modulating vascular smooth muscle cell motility and differentiation [28].

Treatment of endothelial cells with trophoblast-conditioned medium stimulates the endothelial production of CCL5. This chemokine, along with CXCL8 (IL-8) and CCL2, enhances trophoblast cell migration and the invasive properties of trophoblast-derived endothelial cells [29]. Endovascular trophoblasts capture circulating maternal platelets whose granules contain CCL5. Upon release, CCL5 attracts CCR1-positive cells from the cytotrophoblastic shell into the spiral artery and enhances trophoblastic arterial infiltration [30].

Within the complex signaling network comprising invasive trophoblasts, endothelial cells, and leukocytes, extravascular trophoblasts secrete IL-6 and CXCL8, which stimulate endothelial cells to secrete CXCL6 and CCL14. These chemokines subsequently regulate uterine NK cell and macrophage functions [31].

The chemokine CXCL12 promotes tissue neovascularization by recruiting endothelial progenitor cells. CXCL12 binding to CXCR4 initiates a signaling cascade that elevates VEGF levels and enhances VEGF-induced vascularization. Increased VEGF expression creates a positive feedback loop, further amplifying CXCL12 and CXCR4 production. Moreover, the CXCL12/CXCR4 axis interacts with other angiogenic factors—Fgf-2, angiopoietin-1, PLGF, and insulin-like growth factor—enhancing the recruitment of endothelial progenitor cells to placental vascularization sites through CXCR4 binding [32].

**Figure 2:**
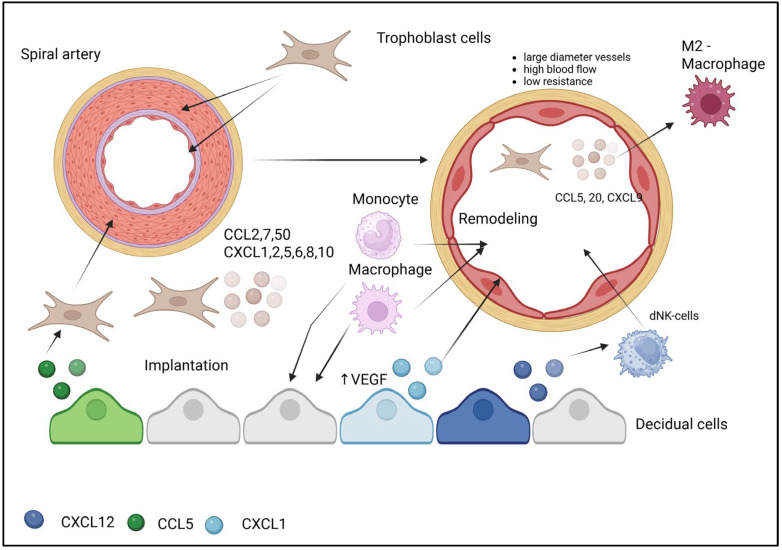
Role of chemokines in spiral artery remodeling.

CXCL1 (growth-related oncogene-α, GRO-α) actively participates in angiogenesis [33]. The CXCL1 gene is robustly expressed in human endometrial stromal cells exposed to trophoblast-conditioned medium [34] and in human uterine stromal fibroblasts treated with decidual lymphocyte factors [35]. Vascular endothelial cells abundantly express CXCR2, the receptor for CXCL1, and CXCL1 presence enhances proliferation, migration, and active vessel formation in HUVEC endothelial cells in vitro. CXCL1 functions through the direct or indirect regulation of VEGF-A expression during angiogenesis. Furthermore, monoclonal antibody-mediated blockade of CXCL1 production in mice reduces uterine size and vascularization during pregnancy [36] (Figure 2).

The transformation of narrow, high-resistance spiral arteries into wide, low-resistance vessels is driven by a coordinated cellular cascade orchestrated by specific chemokines. The process initiates as extravillous trophoblasts (EVTs), guided by CCL5, infiltrate the arterial wall. Simultaneously, CXCL12 recruits decidual NK cells (dNK), which support the remodeling process. Infiltrating EVTs secrete a cocktail of chemokines (CCL2, CCL7, CXCL8) to recruit monocytes from the circulation. Within the vascular niche, these monocytes polarize into M2 macrophages under the influence of CCL5, CCL20, and CXCL9. The M2 macrophages then execute key effector functions: phagocytosing cellular debris and promoting vascular smooth muscle cell dedifferentiation, facilitating their displacement. Concurrently, CXCL1 stimulates angiogenesis by activating VEGF, ensuring adequate vascularization of the remodeled tissue. This sequential recruitment and activation of immune and stromal cells ensures the physiological transformation of maternal vessels to support placental perfusion.

## 5. Chemokines in Implantation and Placentation During Pregnancy

Each developmental stage requires a unique microenvironment providing support and protection. Implantation and early placentation necessitate an inflammatory milieu, while fetal growth is associated with immune tolerance requiring anti-inflammatory signals, and finally, parturition and placental rejection demand a proinflammatory environment [37]. During early pregnancy, coordinated embryonic and endometrial development is crucial for successful implantation and placentation. These processes are tightly regulated through autocrine, paracrine, and endocrine interactions involving sex steroid hormones, growth factors, cytokines, and chemokines that mediate uterine and trophoblast cell proliferation, migration, invasion, and differentiation [14,38].

Embryo implantation and subsequent placentation require balanced hormonal, cytokine, and chemokine interactions between fetus and mother (Figure 1). Disruption of trophoblast invasion and placental cell remodeling at 5–6 weeks of gestation can lead to serious complications including first-trimester miscarriage, membrane rupture, preterm labor, fetal growth restriction, and third-trimester preeclampsia [39]. Trophoblast invasion represents a pivotal event in implantation and placentation, responsible not only for blastocyst embedding but also for placental attachment and the remodeling of uterine spiral arteries to accommodate pregnancy demands [40,41]. This process is regulated by multiple chemokines.

CX3CL1 (fractalkine) and its receptor CX3CR1 are abundantly expressed in reproductive tissues including the ovaries, fallopian tubes, uterus, and testes [41,42,43]. Fractalkine-deficient mice maintain fertility without behavioral abnormalities or brain pathology [43]. Uterine epithelial cells secrete fractalkine during implantation, and the fractalkine/CX3CR1 axis plays a key role in stimulating extravillous trophoblast migration and invasion, although CX3CR1 is absent in interstitial extravillous trophoblasts that migrate through decidual stroma without vascular penetration [44]. Besides glandular epithelial cells, fractalkine is also expressed by decidualized stromal cells, uterine NK cells, and decidual macrophages that may paracrinally modulate invasive trophoblasts. As an adhesion molecule, fractalkine stabilizes interactions between maternal CX3CR1+ leukocytes and the syncytiotrophoblast. In pregnancy pathologies like gestational diabetes or preeclampsia, enhanced fractalkine-mediated leukocyte–syncytiotrophoblast adhesion may activate leukocytes and exacerbate preeclampsia progression, while such adhesive interactions are absent in normal pregnancies [44].

The 8 kDa CXC chemokine CXCL12 (stromal cell-derived factor-1, SDF-1), originally identified in bone marrow stromal cells, signals through G protein-coupled receptors CXCR4 and CXCR7. The CXCL12/CXCR4/CXCR7 axis regulates embryonic development and maternal–fetal immune balance [45]. Embryo-derived CXCL12 enhances implantation rates and promotes endothelial vessel sprouting in vitro. Intrauterine CXCL12 administration in C57BL/6 mice improves endometrial receptivity by upregulating integrin β3 and osteopontin while inducing endometrial angiogenesis with increased CD31/CD34+ vessels near the epithelial layer [46]. First-trimester human trophoblasts secrete CXCL12 which autocrinely promotes trophoblast proliferation/invasion and paracrinally recruits CD56brightCD16- NK cells to the decidua. Autocrine CXCL12/CXCR4 signaling enhances trophoblast invasiveness and matrix metalloproteinase expression. Additionally, trophoblast-derived CXCL12 paracrinally upregulates CD82 in decidual stromal cells, which suppresses excessive trophoblast invasion by inhibiting integrin β1/MAPK/ERK1/2 signaling [47]. Although the critical role of this axis is established, the precise spatiotemporal dynamics of the CXCL12 gradient in vivo and the functional interplay between CXCR4 and CXCR7 in human pregnancy remain to be fully elucidated.

## 6. Chemokine Regulation of Implantation and Pregnancy Maintenance

On day 8 following intrauterine CXCL12 infusion in mice, unsupervised hierarchical cluster analysis revealed 1724 differentially expressed genes (985 upregulated and 739 downregulated) in CXCL12-treated groups, encompassing biological processes including estradiol response, positive regulation of vasodilation, cell adhesion, cell surface receptor signaling, integral components of plasma membrane, extracellular matrix organization, chemokine/cytokine activity, signal transducer activity, protein homodimerization, and vitamin D binding. Gene ontology enrichment analysis demonstrated CXCL12-mediated regulation of key reproductive processes: embryo implantation, sprouting angiogenesis, gonadotropin-releasing hormone secretion, blood vessel development, epidermal growth factor receptor signaling pathway, collagen metabolic processes, and muscle contraction regulation [46]. Elevated CXCL12 levels in follicular fluid correlate with corpus luteum formation and promote both follicular development and luteinization [48], while follicular fluid cytokines (IFN-γ, IL-10, IL-12, and TNF) involved in granulosa cell proliferation, oocyte viability, and ovulation [49] represent potential targets of CXCL12-CXCR4 luteal activity. Increased levels of IFN-γ, IL-10, IL-12, TNF, CXCL12, and CXCR4 during the luteal phase may reflect altered synthesis patterns by steroidogenic luteal cells, with CXCL12 specifically stimulating cytokine production rather than progesterone synthesis, as evidenced by increased TNF secretion but unchanged progesterone output in CXCL12-treated steroidogenic cells [50]. Ovine placental studies demonstrate predominant CXCL12 localization in endometrial stroma, glands, and trophoblasts, with peak immunoreactivity in trophoblasts at gestational day 22 following natural conception, suggesting CXCL12 signaling influences embryo viability and pregnancy establishment, while impaired CXCL12 signaling in ART-derived embryos may contribute to adverse pregnancy outcomes [51]. Although CXCL12 exhibits higher binding affinity for CXCR7 than CXCR4, functional studies reveal that CXCR4 predominates in human trophoblasts and decidual epithelial cells, mediating CXCL12’s primary effects through differential temporal expression patterns—late-gestation trophoblasts show diminished CXCL12 production compared to first-trimester cells, though secretion remains stable for ≥96 h in vitro, suggesting stage-specific regulatory roles. Recombinant human CXCL12 (rhCXCL12) promotes trophoblast proliferation and inhibits apoptosis, effects attenuated by anti-CXCL12, -CXCR4, or -CXCR7 antibodies [52,53]. The chemokine CCL2 (MCP-1), encoded on chromosome 17q11.2 [54], is secreted by the first-trimester human decidua via ERK/MAPK signaling [55] under regulation by estrogen, progesterone, and hCG. CCL2 drives the Th2 polarization of naïve T-cells in vitro, with 55% of decidual NK cells, 52% of CD4+ T-cells, and 75% of CD14+ monocytes expressing CCR2 (CCL2 receptor). Recombinant CCL2 and decidual stromal supernatants enhance decidual leukocyte proliferation/survival and Th2 cytokine (IL-4, IL-10) production via GATA-3 upregulation [56], while estradiol-stimulated NK cell-derived CCL2 creates an autocrine loop supporting NK cell maintenance in the decidua [57]. As a crucial recruiter of Th2 cells, non-cytotoxic NK cells, Tregs, and myeloid-derived suppressor cells to the utero-placental interface, CCL2 deficiency in early pregnancy may disrupt the Th2/Treg balance required for subsequent gestational immune homeostasis [58].

During early pregnancy, macrophages at the maternal–fetal interface exhibit a unique mixed M1/M2 phenotype regulated through autocrine and paracrine mechanisms, with the CCR2-JAK2-STAT3 signaling axis being essential for maintaining decidual macrophage homeostasis in vivo. Pharmacological inhibition of JAK2 significantly reduces decidual macrophage populations while decreasing the expression of proliferation marker Ki67, scavenger receptor CD36, costimulatory molecule CD86, mannose receptor CD206, and proinflammatory cytokines TNF and IL-1 [59]. These infiltrating macrophages support pregnancy through three key mechanisms: promoting angiogenesis, facilitating placental development, and establishing immune tolerance. The physiological hypoxia characteristic of first-trimester maternal–fetal interface enhances macrophage recruitment and adhesion via hypoxia-induced upregulation of CCL2 and stromal ICAM2/ICAM5 expression in a VEGFA-dependent manner [60]. Elevated placental CCL2 during early gestation activates CD14+ myelomonocytic cells, with coculture experiments demonstrating that HTR8/SVneo trophoblasts synergize with these cells to produce >100-fold higher CCL2 levels compared to monocultures, where CD14+ cells serve as the primary CCL2 producers while trophoblasts facilitate production through direct cell–cell contact. CCL2 further regulates immune tolerance by controlling arginase-1 expression and expanding immunosuppressive CD14+HLA-DR−/low myeloid precursors [61].

The CXC chemokine ligand 16 (CXCL16) exists in two functionally distinct isoforms: a transmembrane form functioning as both a scavenger receptor for oxidized LDL (oxLDL) and an adhesion molecule for CXCR6-expressing cells, and a soluble form generated through proteolytic cleavage that mediates leukocyte homing [62]. At the maternal–fetal interface, CXCL16 demonstrates robust expression and participates in cytotrophoblast invasion and placental development [63]. Immunohistochemical studies reveal CXCL16 presence in the cytoplasm and plasmalemma of villous cytotrophoblasts, syncytiotrophoblasts, and extravillous trophoblasts during first-trimester human pregnancy, with additional expression in decidual stromal cells, endometrial stromal cells, and decidual immune cells [64]. Trophoblast-derived CXCL16 promotes endometrial decidualization through PI3K/PDK1/AKT/Cyclin D1 pathway activation and induces M2 macrophage polarization in vitro. These polarized M2 macrophages subsequently regulate NK cell phenotype by downregulating IL-15 expression, thereby reducing NK cytotoxicity and establishing maternal–fetal immune tolerance. CXCL16 also functions as a pro-angiogenic cytokine stimulating spiral artery remodeling via ERK signaling [40]. Receptor distribution studies show high CXCR6 expression on peripheral monocytes and γδT cells, moderate levels on T cells and NKT cells, and low expression on both CD56+CD16− and CD56+CD16+ NK cell subsets [65]. CXCL16 induces dose-dependent trophoblast proliferation and invasion, demonstrating stronger effects than CXCL12, while also reducing decidual cell granzyme B secretion and upregulating anti-apoptotic Bcl-xL in trophoblasts [66]. These findings collectively position the CXCL16-CXCR6 axis as a critical regulator of early placental development and immune tolerance establishment.

In early human pregnancy, fetal trophoblasts come into direct contact with maternal immune cells at the maternal–fetal interface. Fetal trophoblasts can guide the migration of maternal T-lymphocytes and monocytes into the decidua by secreting CXCL16. The sole receptor for CXCL16, CXCR6, is predominantly expressed in T-lymphocytes, NKT cells, and monocytes but shows negligible expression in the two NK-cell subsets from peripheral blood or decidual tissue. The CXCL16/CXCR6 interaction mediates the migration of peripheral T-lymphocytes, γδ-T cells, and monocytes—but not NKT cells. Under the influence of trophoblast-conditioned medium, peripheral blood cells differentiate into a leukocyte population resembling the decidual lymphocyte composition [65].

Endometrial remodeling occurs cyclically during the estrous cycle and pregnancy, coinciding with changes in the uterine cavity epithelium, stroma, and vascular endothelium. When endogenous chemokines are highly expressed, their specific receptors (e.g., CCR1, -2, -3, -5, and CXCR2) may relocate to the cytoplasm and cell nuclei. In contrast, chemokines with low expression failed to induce receptor internalization, leaving receptors membrane-bound (e.g., CXCR3, -4). Ligand-induced internalization of G protein-coupled receptors is a common phenomenon, which may either downregulate signaling by removing active receptors from the membrane or sustain signaling initiated at the plasma membrane [67].

CCL8, expressed by endometrial cells, contributes to endometrial preparation for embryo contact. While chemokines collectively foster a suitable microenvironment for implantation, they may also negatively regulate endometrial/embryo adhesive properties. In pigs, trophoblasts with upregulated CCL8 expression on embryonic day 12 recruit epithelial cells to the trophoblast-occupied area and enhance epithelial folding. Endometrial cells cultured in stable CCL8 concentrations exhibit higher viability and tend to form an intact monolayer [68].

**Figure 3:**
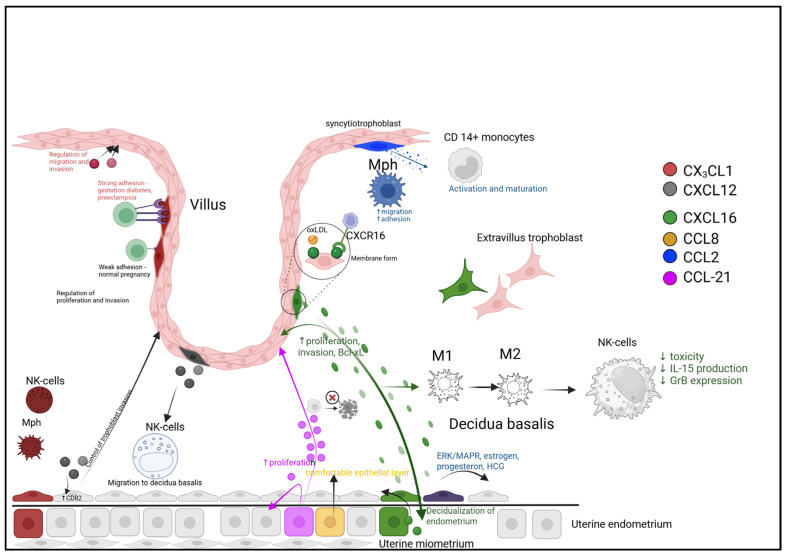
Chemokine networks fine-tune the immune microenvironment at the maternal-fetal interface.

The small chemokine CCL21 belongs to the CC-chemokine family and binds to CCR7 to mediate intracellular signaling [69]. CCL21 contains a domain with conserved cysteine residues that is structurally similar to secreted growth factors and interferons involved in mitogenic, chemotactic, and inflammatory activities. Its structure features two highly conserved disulfide bonds and demonstrates significant homology with CXCL8, another member of the small chemokine family [70]. The CCL21-CCR7 signaling pathway is essential for trophectoderm development and endometrial epithelial differentiation, as well as for regulating external factors that contribute to endometrial infections and stress, and for successful embryo implantation and pregnancy maintenance in pigs. During early pregnancy in pigs, the expression of CCL21 and its receptor CCR7 increases in endometrial epithelial cells. CCL21 stimulates the proliferation of trophoblast and endometrial cells, suppresses endoplasmic reticulum stress-induced apoptosis, and inhibits LPS-induced inflammation. These regulatory effects of CCL21 are mediated through the activation of the PI3K/AKT and MAPK signaling pathways, along with inhibition of the UPR and TLR4/NF-κB pathways [71] (Figure 3).

Successful implantation and early placentation require a precisely balanced immune environment, established through distinct chemokine-driven pathways. The schematic illustrates three key regulatory mechanisms:

(1) Cellular Recruitment and Education. Trophoblast-derived CXCL16 acts via CXCR6 to recruit maternal T-cells and monocytes, shaping the decidual leukocyte composition. The CCL21/CCR7 axis promotes trophoblast and endometrial cell proliferation while suppressing stress-induced apoptosis, thereby enhancing tissue viability.
(2) Macrophage Polarization Balance. The local microenvironment, influenced by factors like oxidized LDL (oxLDL), drives macrophage differentiation. A balance between pro-inflammatory (M1, potentially modulated by CCL2) and regenerative (M2, modulated by CCL18/CCL22) phenotypes is crucial for immune tolerance and tissue repair.
(3) Spatial and Dynamic Regulation. Chemokines are strategically localized, with CCL8 enriched at the implantation zone to support epithelial reorganization, and CCL21 at the trophectoderm interface. Furthermore, ligand-induced receptor internalization (e.g., CCR1/CCR5) provides a dynamic feedback mechanism to attenuate signaling, while persistently membrane-bound receptors (e.g., CXCR3/CXCR4) may sustain chemotactic gradients.

The second immunological phase of pregnancy, characterized by rapid fetal growth and development, represents an optimal physiological period for the mother. During this phase, the mother, placenta, and fetus establish symbiotic relationships, with the predominant immunological feature being the induction of an anti-inflammatory state [72].

With the transition to the second trimester, IL-1β and TNF-α stimulate macrophages to produce chemokines that recruit dendritic cells through MAPK and NF-κB signaling pathways. CCL2 (MCP-1) serves as the primary chemoattractant for macrophages, while CCL5 (RANTES) predominantly recruits immature dendritic cells [73].

As pregnancy progresses, regulatory mechanisms are activated to restrain excessive trophoblast invasion. This process is associated with decreased levels of chemokines IP-10 (CXCL10), MCP-1 (CCL2), and MIP-1β (CCL4) during the second trimester. Disruption of these regulatory pathways may lead to placental abnormalities and pregnancy loss [74].

During the second trimester of pregnancy, erythroid cells constitute one of the most abundant cellular populations and function as active producers of various chemokines including CCL2, CCL3, CCL4, and CXCL1 [75]. Emerging evidence indicates that erythroid cells demonstrate remarkable similarity in their chemokine secretion profiles to multiple cell populations at the maternal–fetal interface. These cells exhibit parallel chemokine expression patterns with decidual stromal cells, trophoblasts, decidual macrophages, and dendritic cells, suggesting their potential involvement in establishing the unique immunological microenvironment required for a successful pregnancy [55,76,77,78]. Both human and murine pregnancies demonstrate enhanced erythropoiesis with increased numbers of CD71+ erythroid cells that possess immunosuppressive properties [79]. These pregnancy-associated erythroid cells continuously produce TGF-β, a cytokine that promotes the generation of regulatory T cells while simultaneously suppressing Th17 cell induction [80]. Consequently, the second trimester is characterized by active recruitment of immunoregulatory cells to the decidua, where the migrated cells are maintained in an immunosuppressive state to preserve the anti-inflammatory environment and sustain feto-maternal tolerance. This intricate immunomodulatory process involves coordinated action between erythroid-derived chemokines and their cellular targets, creating a specialized immunological niche that supports ongoing placental development while protecting the semiallogeneic fetus from maternal immune rejection. The expanded population of immunosuppressive CD71+ erythroid cells appears to play a pivotal role in maintaining this delicate balance through both chemokine-mediated recruitment and direct cytokine-mediated suppression of proinflammatory responses.

## 7. Chemokines in the Regulation of Labor

The anti-inflammatory response characteristic of early pregnancy, essential for supporting implantation, gives way to processes of tolerance and regeneration to facilitate fetal development. However, at the end of pregnancy, a proinflammatory state re-emerges to overcome this tolerance and initiate labor [37]. During late pregnancy, the decidua and activated decidual leukocytes trigger an inflammatory cascade that culminates in the production of large quantities of prostaglandins, which enhance myometrial activation and contractility. The myometrium regulates the synthesis and release of cytokines that provide strong chemotactic signals for leukocyte infiltration [81].

Labor involves coordinated interactions between multiple maternal and fetal cell types, including myofibroblasts, trophoblasts, endothelial cells, and immune cells [82]. Single-cell RNA sequencing (scRNA-seq) analysis has revealed that monocytes/macrophages are the most dynamically altered immune cell subsets in uterine tissues during term labor [83].

During labor, numerous signaling pathways and chemokine/chemokine receptor systems are activated in the decidua, accompanied by an increased expression of inflammatory mediators. CCL2 (MCP-1) and M-CSF play key roles in mobilizing monocytes from the bone marrow into the bloodstream. The concentrations of CCL2 and M-CSF are significantly higher in umbilical cord blood than in peripheral blood during labor, suggesting that trophoblasts and decidual stromal cells at the feto-placental interface are the primary sources of these factors. Local production of these mediators may stimulate bone marrow activation and monocyte release. Syncytiotrophoblasts produce chemotactic factors such as MIF and CXCL10 (IP-10), which recruit immune cells, including mucosal-associated invariant T cells (MAITs), B cells, and effector memory T cells (TEM), thereby maintaining the placenta’s unique immunological niche [84].

Elevated chemokine concentrations represent one of the key mechanisms driving labor [85]. Cytokine–receptor interactions may be shared between spontaneous preterm labor and miscarriages, involving 27 differentially expressed genes (DEGs) in preterm labor and 26 DEGs in miscarriages. Network analysis highlights the central role of DEGs predominantly belonging to the CC and CXC chemokine subfamilies, including CXCR4. Most DEGs show upregulated expression, except for CCR7, which is downregulated in miscarriages. The CXCR3 ligands CXCL9, CXCL10, and CXCL11 are elevated in spontaneous preterm labor, while CXCL10 is also increased in miscarriages [86].

During the third trimester of pregnancy, the myometrium and decidua secrete numerous proinflammatory cytokines, chemokines, and growth factors that promote the onset of labor. The decidua contains significantly higher levels (10–1000 times greater) of 19 key chemokines, proinflammatory cytokines, and growth factors compared to the myometrium. These cytokines play important roles in leukocyte migration to the uterus, acting as chemoattractants for monocytes (CCL2, CCL4, CXCL10, and CX3CL1), neutrophils (CXCL1 and CXCL8), or lymphocytes (CCL5 and CXCL16). The inflammatory state in uterine tissues during labor leads to a significant increase in CXCL8 and CCL2 [87]. The highest level of inflammation, associated with IL-6 and CXCL8, was observed in women with term labor compared to those with preeclampsia or preeclamptic syndrome. Additionally, both term and preterm labor showed elevated levels of the Th2-associated chemokine CCL17, suggesting that the inflammatory process during labor is more complex than a simple Th1/Th2 switch [88]. Chemokines such as HCC-4 (CCL16), I-TAC (CXCL11), MIP-3α (CCL20), and TARC (CCL17) may significantly contribute to the pathogenesis of preterm labor [89]. CCL20 (Exodus-1, macrophage inflammatory protein-3α (MIP-3α)) is a chemokine that interacts with the chemokine receptor CCR6 [90] and shares properties with certain antimicrobial peptides like β-defensins [91]. CCL20 is present in amniotic fluid, and its concentration increases as labor approaches. Spontaneous labor may be associated with increased bioavailability of CCL20, which represents part of the general human labor mechanism [92]. During term labor, there is increased expression of chemokines CCL2, CCL4, CCL5, CXCL8, and CXCL10 at both mRNA and protein levels, while CCL8 is elevated only during preterm labor. These chemokines act as chemoattractants for monocytes/macrophages, consistent with the increased number of macrophages in the decidua during labor, which participate in the labor process through local activation and amplification of inflammatory responses. In addition to recruiting monocytes and macrophages, these chemokines attract T cells, NK cells, and dendritic cells [93]. Pregnancy loss, stillbirth, and spontaneous preterm labor share many key features and risk factors. Epigenetic regulation of the chemokine–cytokine pathway, including coding and long non-coding RNAs involved in the pathogenic mechanism of cytokine– and chemokine–receptor interactions, may represent a common epipathogenic mechanism associated with various adverse pregnancy outcomes. In spontaneous miscarriage compared to labor, the three most common biological processes were monocyte chemotaxis, leukocyte migration, and molecular functions, namely, receptor–ligand activity, signaling receptor binding, and chemokine receptor binding [86].

Inflammation in the myometrium plays a crucial physiological role in transitioning the uterine muscle from a quiescent to a contractile state during labor. The myometrium becomes infiltrated with neutrophils, macrophages, and T-lymphocytes that serve as primary sources of inflammatory cytokines, while the expression of chemokines and endothelial adhesion molecules increases significantly with labor onset, suggesting that these mediators facilitate cellular infiltration into the myometrial tissue [94]. Peripheral blood leukocytes exhibit early chemotactic responses during late pregnancy that enable their migration into uterine tissues. Monocytes are initially recruited to the myometrium by various cytokines and chemokines, but following transmigration, activated monocytes paradoxically limit further chemotaxis by disrupting local CCL2 concentration gradients [95]. Mechanical stress from the growing fetus elevates CCL2 levels, an effect replicated in vitro through the stretching of cultured myometrial cells [96]. Myometrial cell stretching also increases IL-8 expression and release primarily through NF-κB-dependent mechanisms, with human myometrial cells showing peak expression of CXCL1 and CXCL8 six hours after mechanical stimulation [97].

The key chemokines mediating inflammatory processes during labor are CXCL8 and CCL2, acting through their respective receptors CXCR2 and CCR2. CXCL8 serves as a potent neutrophil chemoattractant with elevated mRNA expression in myometrium during both term and preterm labor, while CCL2 (MCP-1) produced by decidual, endometrial, and myometrial cells effectively recruits macrophages to cervical, myometrial, and fetal membrane tissues at labor onset [95]. Decidual tissues from women with chorioamnionitis-induced preterm labor show substantial infiltration of maternal neutrophils recruited by decidual-derived chemokines like CXCL8 [98]. During normal labor, T-cells are attracted to sites of fetal membrane rupture primarily through CXCL10- and CCL5-mediated chemotaxis, though this recruitment is significantly diminished in cases of preterm premature rupture of membranes [99]. Acute chorioamnionitis as a major cause of preterm labor associates with elevated T-cell chemokines (CXCL9, -10, and -11) in umbilical cord blood, while cytotoxic T-cells expressing chemokine receptors (CXCR3 and CCR5) are prominently present in villitis of unknown etiology [100].

In the reproductive system, CCL2 production by trophoblasts, decidual cells, and endometrial/myometrial cells increases markedly during pregnancy—detectable in the mouse uterus on gestation day 1 and the human myometrium during late pregnancy. Rat myometrium shows significant upregulation of Ccl-2 gene and protein expression before and during labor, suggesting positive regulation by mechanical stretching from fetal growth and negative regulation by progesterone withdrawal. CCL2 appears to integrate mechanical and endocrine signals promoting uterine inflammation and labor initiation, as mechanical stretch increases its expression in human mesangial and endothelial cells. During late pregnancy, combined hormonal and mechanical stimuli enhance Ccl-2 expression and secretion by uterine smooth muscle cells, creating chemotactic gradients that attract circulating CD68-positive monocytes from local blood flow [101]. Activated macrophages subsequently release matrix metalloproteinases (facilitating cervical ripening and membrane rupture), prostaglandins/histamine/serotonin (inducing direct uterine contractions), and cell adhesion molecules [102], collectively executing the final processes of parturition through this carefully coordinated inflammatory cascade. Dysregulation of these chemokine-mediated pathways may contribute to pathological conditions like preterm labor, highlighting their importance in both normal and complicated obstetric outcomes.

Beyond stimulating labor, CCL2 induction in the myometrium may also represent a regulatory mechanism for postpartum uterine involution. Uterine involution involves substantial tissue reorganization, matrix metalloproteinase induction, extracellular matrix degradation, and apoptosis. CCL2 production could significantly accelerate these processes [101]. Other chemokines that play important roles in inflammatory endometrial breakdown during menstruation (MCP-3, eotaxin, FNK, and MIP-1β) [103] may also contribute to postpartum decidual tissue breakdown and myometrial involution.

The myometrial transcriptome during spontaneous term labor differs dramatically from that of non-laboring women, with differential expression of over 400 genes between groups. Overall, spontaneous term labor is characterized by a molecular signature consistent with the overexpression of genes involved in inflammation and leukocyte chemotaxis. During labor, chemokine signaling pathways become activated in the myometrium. Interleukin-8 serves as the primary chemokine mediating neutrophil chemotaxis and activation. Intrauterine IL-8 production is regulated by progesterone, and progesterone withdrawal near term may drive increased IL-8 production, neutrophil infiltration and activation, and the subsequent myometrial inflammatory phenotype.

Spontaneous term labor demonstrates upregulated expression and production of CCL2, a monocyte chemoattractant also involved in macrophage activation. Progesterone suppresses CCL2 expression in pregnant rat myometrium, making CCL2 a potential therapeutic target for preventing preterm labor [104]. CXCL6, induced by IL-1β [105], shares high functional homology with IL-8 as a potent neutrophil chemoattractant and is overexpressed in chorioamniotic membranes during labor [85]. Both mRNA and protein expression of CXCL6 are significantly elevated in the human myometrium during spontaneous term labor without histological chorioamnionitis.

**Figure 4:**
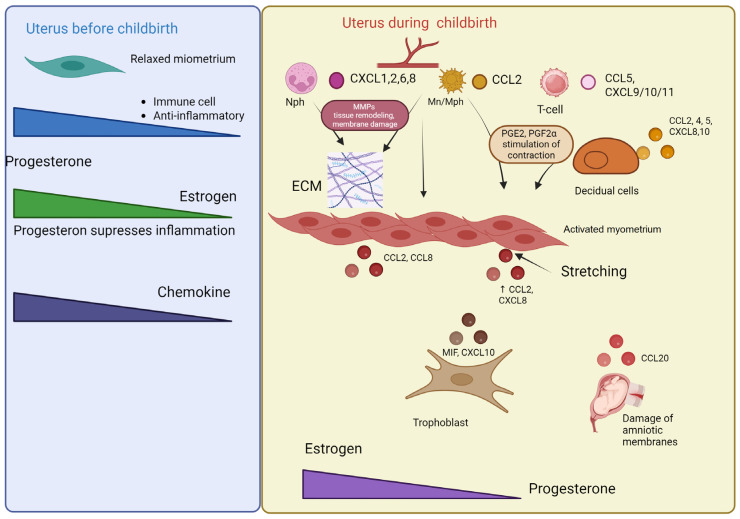
Chemokine-driven inflammatory cascade initiates parturition.

This coordinated chemokine activity not only initiates labor but appears to continue regulating tissue remodeling processes postpartum. The transition from pregnancy maintenance to labor and subsequent involution represents a continuum of carefully regulated inflammatory processes mediated by specific chemokine networks. The differential expression patterns between laboring and non-laboring states highlight the profound molecular reprogramming required for successful parturition and postpartum recovery. These findings suggest chemokines serve dual roles in both initiating labor and facilitating postpartum uterine remodeling through similar inflammatory mechanisms (Figure 4).

The transition from uterine quiescence to active labor is mediated by a fundamental shift in the chemokine landscape. During pregnancy (quiescence), progesterone dominance suppresses pro-inflammatory chemokine production, minimizing immune cell infiltration and maintaining myometrial relaxation. At term (labor), this balance is overturned. A pro-inflammatory cascade is triggered, leading myometrial, decidual, and trophoblast cells to secrete a broad spectrum of chemokines that establish distinct recruitment gradients. CCL2 and CCL4 attract monocytes and macrophages. CXCL8, CXCL1, CXCL2, and CXCL6 act as potent chemoattractants for neutrophils. CCL5, CXCL9, CXCL10, and CXCL11 facilitate the recruitment of T-lymphocytes.

This massive immune cell infiltration amplifies the inflammatory response. Critically, infiltrating macrophages release prostaglandins that directly stimulate myometrial contractions and matrix metalloproteinases (MMPs) that remodel extracellular matrix (ECM) and promote fetal membrane rupture. Mechanical stress from the growing fetus further amplifies the production of CCL2 and CXCL8, creating a positive feedback loop that locks the uterus into the labor process.

## 8. Preterm Birth: Chemokine-Mediated Mechanisms and Pathophysiology

Preterm birth (delivery before 37 weeks of gestation) remains a leading cause of infant mortality and morbidity worldwide, independent of a nation’s economic development status [106]. The pathophysiology of preterm labor involves complex mechanisms that prematurely activate inflammatory cascades, primarily mediated through intrauterine infection, hemorrhage, uterine overdistension (multiple gestation and polyhydramnios), and maternal–fetal stress [107].

The decidua serves as a crucial immunomodulatory interface, transmitting signals from aging fetal membranes to the myometrium. This process is characterized by abundant activated innate immune cells within the decidual layer that secrete potent proinflammatory cytokines/chemokines, prostaglandins, and matrix metalloproteinases [108,109]. Human decidual tissues demonstrate significantly higher macrophage density during term labor compared to preterm gestation without labor, correlating with elevated levels of the monocyte–macrophage chemoattractant CCL2 in both term and preterm labor [93].

Transcriptomic analyses reveal that monocytes from women undergoing active preterm labor exhibit differential expression of over 40 genes compared to term labor controls, with particular alterations in chemokines CXCL2 and CXCL8 and their receptors. Peripheral blood monocytes and neutrophils are recruited to uterine tissues through cytokine/chemokine gradients, with fetal membrane extracts from women experiencing Braxton Hicks contractions inducing 36-fold higher monocyte chemotaxis and 3-fold higher neutrophil chemotaxis compared to non-laboring controls [110].

The human myometrium produces substantial quantities of chemokines including CCL2, CCL7, and CCL20. Proinflammatory cytokines IL-1β and TNF-α significantly enhance chemokine expression specifically during labor initiation rather than late pregnancy, representing a protective mechanism to prevent excessive tissue damage [111]. The transition from myometrial quiescence to contractility involves a fundamental shift from anti-inflammatory to proinflammatory signaling pathways, characterized by increased production of interleukin-8, interleukins-1 and -6, and contraction-associated proteins (oxytocin receptors, connexin-43, and prostaglandin receptors). Throughout gestation, progesterone maintains myometrial quiescence by suppressing the expression of both contraction-associated proteins and inflammatory cytokines/chemokines [112].

In infection-mediated preterm labor with chorioamnionitis, neutrophil chemokines CXCL-8, CXCL1, and CXCL2 demonstrate consistent elevation across all uterine tissues, while monocyte chemokines show tissue-specific patterns: CCL2 increases in both the choriodecidua and amnion, whereas CCL5 elevation is restricted to the choriodecidua. During preterm premature rupture of membranes with infection, maternal innate immune activation increases neutrophil chemokine levels, though specific neutrophil responses vary according to bacterial type and tissue microenvironment [113].

Notably, women with spontaneous preterm labor and intra-amniotic infection show no significant differences in amniotic fluid concentrations of CXCL10 and CXCL11 compared to controls [113]. In both bacterial and sterile intra-amniotic inflammation, MIP-1α emerges as the most significantly involved chemokine in the inflammatory process [114].

Single-cell transcriptomic analysis of human amnion reveals dramatic CCL20 upregulation at the rupture site, with CCL20 representing one of the most actively regulated genes across all major amniotic cell types during labor. The functional significance of this finding is confirmed by intraperitoneal CCL20 injection in mice, which induces preterm birth, underscoring this chemokine’s crucial role in labor initiation [115].

The chemokine receptor CXCR3, which binds CXCL9 and CXCL10, is present in human amniotic fluid during the third trimester. Women with preterm birth associated with chronic placental inflammatory lesions exhibit elevated amniotic fluid concentrations of CXCL9 and CXCL10, reflecting pathological chronic inflammation. In contrast, spontaneous term labor does not increase CXCR3, CXCL9, or CXCL10 levels in amniotic fluid [116]. The CXCL9/CXCR3 signaling pathway regulates maternal immune cell trafficking to the maternal–fetal interface and may facilitate maternal–fetal tolerance. Disruption of this pathway is associated with fetal demise and preterm birth mechanisms, paralleling its established role in transplant allograft rejection [117].

Elevated placental CX3CL1 expression is associated with several pregnancy complications including chorioamnionitis, gestational diabetes, and preeclampsia. Experimental studies demonstrate that CX3CR1 deletion or anti-CX3CL1 antibody administration prevents preterm delivery in mice. Increased CX3CL1 enhances leukocyte influx and promotes proinflammatory macrophage polarization at the maternal–fetal interface, resulting in shortened gestation. Early pregnancy CX3CL1 measurement predicts the risk of premature rupture of membranes with 90% sensitivity and 40% specificity, highlighting its potential as a clinical biomarker [117].

## 9. Chemokines in Pregnancy Pathologies: Preeclampsia

**Figure 5:**
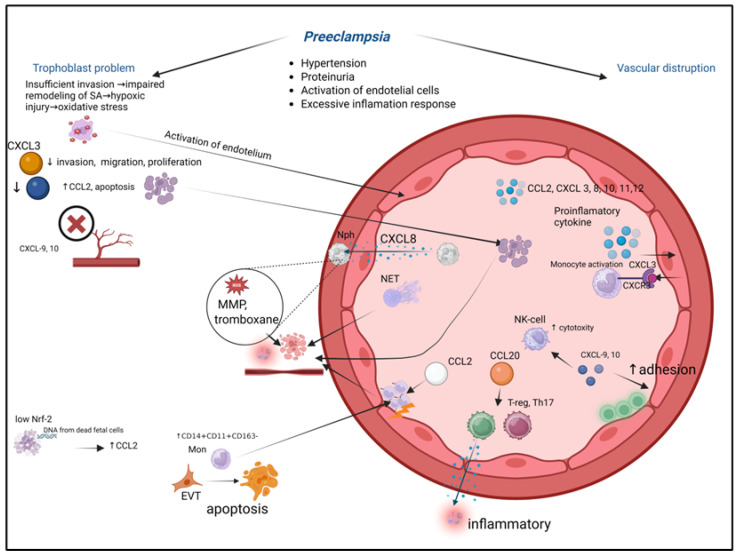
Chemokine dysregulation as a central driver of preeclampsia pathogenesis.

Preeclampsia (PE) is a serious and life-threatening pathological condition during pregnancy that affects 2–4% of pregnant women worldwide [118]. Preeclampsia is characterized by gestational hypertension, proteinuria, systemic endothelial cell activation, and an excessive inflammatory response [119]. The pathogenesis of preeclampsia may be associated with inadequate trophoblast invasion, leading to endothelial dysfunction and an exaggerated inflammatory reaction [120]. Insufficient trophoblast invasion results in impaired remodeling of spiral arteries, hypoxic-reperfusion injury, and oxidative stress in the placenta, which stimulates the release of substances from the syncytium that activate the maternal endothelium [121,122]. Preeclampsia is linked to the disruption of feto-maternal tolerance [123] (Figure 5). 

Preeclampsia arises from a pathological cascade that originates in the placenta and propagates to the maternal systemic circulation, with chemokine dysregulation serving as a critical link. The process depicts key mechanistic steps:

(1) Placental Insult. Shallow extravillous trophoblast (EVT) invasion fails to adequately remodel maternal spiral arteries, causing placental hypoxia and oxidative stress.
(2) Chemokine Storm. The stressed placenta releases a surge of pro-inflammatory chemokines, including CCL2, CXCL8, and CXCL10.
(3) Immune Activation and Vascular Dysfunction. These placental chemokines recruit and activate maternal neutrophils and monocytes into the intervillous space and systemic circulation. Activated immune cells exacerbate local damage, induce endothelial dysfunction, and contribute to a systemic inflammatory response.
(4) Systemic Consequences. The widespread endothelial activation manifests clinically as hypertension and proteinuria. Furthermore, the altered chemokine milieu disrupts the delicate immune tolerance at the maternal-fetal interface, perpetuating the pathological cycle. This figure integrates how localized placental pathology, mediated by chemokine networks, escalates into the multisystemic maternal syndrome of preeclampsia.

The condition represents a complex interplay between impaired placentation, endothelial dysfunction, and systemic inflammation, with chemokines playing a crucial role in mediating these pathological processes. The inadequate transformation of spiral arteries leads to placental ischemia and the release of anti-angiogenic factors and inflammatory mediators into the maternal circulation, triggering widespread endothelial activation and the characteristic clinical manifestations of the disorder. This pathological cascade involves multiple chemokine networks that regulate immune cell recruitment and activation at the maternal–fetal interface, contributing to both the initiation and progression of the disease. The imbalance in chemokine signaling further exacerbates the inflammatory milieu and disrupts the delicate immune tolerance required for successful pregnancy maintenance, ultimately leading to the multisystem manifestations that define preeclampsia.

Numerous studies have demonstrated that patients with severe preeclampsia exhibit elevated plasma levels of various chemokines—CXCL8 (IL-8), CCL2 (MCP-1), CXCL10 (IP10), and CXCL12 (SDF-1)—compared to healthy pregnant women [124,125,126]. Women with preeclampsia show increased circulating levels of CXCL10, CXCL11, CXCL12, and CXCL3 [119]. The development of preeclampsia is associated with higher serum concentrations of CXCL8 (IL-8) and increased expression in placental tissue. CXCL8 contributes to preeclampsia pathogenesis by recruiting neutrophils into vascular intima, where they release reactive oxygen species, myeloperoxidase, MMP-8, and thromboxane, causing cellular damage, endothelial inflammation, and vasoconstriction. Neutrophil extracellular traps formed in placental intervillous spaces enter maternal circulation, promoting thrombosis, systemic inflammation, and fetal demise [127].

The activation-regulated chemokine CCL20 (also known as MIP-3α and Exodus-1) interacts with chemokine receptor CCR6. Binding to CCR6 triggers strong chemotactic responses and intracellular calcium mobilization. CCL20 mediates the recruitment of both proinflammatory Th17 cells and regulatory Tregs to inflammatory sites, suggesting that altered CCL20 expression may disrupt the Treg/Th17 balance in PE. Maternal plasma CCL20 levels are significantly higher in PE patients compared to healthy controls during both early and late pregnancy, though this chemokine normally increases as pregnancy progresses [128].

Severe preeclampsia is characterized by elevated plasma CXCL3 levels but decreased placental CXCL3 expression [129], potentially representing a compensatory mechanism [130]. CXCL3 participates in multiple pathophysiological processes and correlates with cancer progression. Given the similarity between trophoblast and malignant cell invasive properties, CXCL3’s effects on trophoblast invasion may play a key role in preeclampsia pathogenesis. Endogenous downregulation of CXCL3 significantly suppresses the migration, invasion, and proliferation of human trophoblasts, while upregulation enhances these processes. Reduced placental CXCL3 expression leads to inadequate extravillous trophoblast invasion and shallow placental implantation, contributing to severe preeclampsia development [131]. These findings highlight the complex chemokine dysregulation in preeclampsia, involving both systemic inflammatory activation and impaired placental development through distinct molecular mechanisms.

Non-coding RNAs play a crucial regulatory role in trophoblast invasion, extravillous trophoblast differentiation, and spiral artery remodeling—key processes in preeclampsia (PE) development [132]. Among differentially expressed non-coding RNAs, TARID shows significantly reduced expression in PE patients. TARID overexpression markedly enriches the TNF signaling pathway and induces substantial changes in CXCL3 regulation, as CXCL3 is located downstream of TARID and directly regulated by it [133]. CXCL3-mediated migration depends on p38 and ERK1/2 MAPK pathways through CXCR1 and CXCR2 receptors [134], with the ERK1/2 inhibitor PD98059 effectively attenuating CXCL3-induced cell proliferation and migration [135].

Decidual stromal cells from PE patients exhibit elevated CCL5 and CXCL2 expression compared to healthy pregnancies, potentially due to suppressed miR-92a expression in PE-derived cells. miR-92a directly targets IRF3 mRNA, whose overexpression promotes cytokine secretion. PE placentas demonstrate significantly increased M1 macrophage infiltration compared to controls, with miR-92a suppression in decidual stromal cells promoting macrophage polarization while inhibiting trophoblast migration and proliferation [136].

The endothelial-derived chemokine CX3CL1 (fractalkine), activated by proinflammatory cytokines, and its receptor CX3CR1 are expressed on human invasive trophoblasts and associated with placental vascularization [137]. CX3CL1 critically regulates multiple signaling pathways (Nrf2/Keap-1, MAPK, PI3K/AKT, JNK, MEK1/2, NFκB, and JAK2-STAT1) essential for cellular communication, homeostasis maintenance, and responses to physiological/pathological stimuli [137]. As the sole membrane-bound CX3C chemokine, CX3CL1 mediates CX3CR1+ monocyte adhesion to endothelial cells for vascular surveillance. During inflammation, enhanced endothelial CX3CL1 expression promotes firm monocyte adhesion and activation. CX3CL1 demonstrates remarkable diagnostic value, distinguishing early-onset (delivery < 34 weeks) from late-onset PE (delivery ≥ 34 weeks) and showing unique dysregulation in preterm labor through multiplex analysis [138].

Elevated maternal TNF-α may amplify placental CX3CL1 expression/release, exacerbating systemic inflammation in PE. While decidual cell-derived CX3CL1 contributes to PE pathogenesis, circulating CX3CL1 levels lack predictive value. CX3CR1 expression is significantly upregulated in maternal decidua during PE, particularly in decidual macrophages. The FAK signaling pathway, crucial for VEGF-mediated cytotrophoblast migration/invasion, is impaired by high CX3CL1-CX3CR1 levels that suppress VEGF protein expression in M1 macrophages. This inhibits FAK phosphorylation, reduces trophoblast migratory/invasive capacity, and ultimately compromises spiral artery formation through VEGF downregulation and vascular endothelial dysfunction [139]. These findings collectively highlight the complex interplay between chemokine networks, non-coding RNAs, and angiogenic factors in PE pathophysiology.

CX3CL1 release increases during the third trimester primarily through metalloproteinase activity, which is also elevated in preeclampsia. Via its receptor, CX3CL1 upregulates over 30 genes encoding proteins involved in trophoblast migration, including extracellular matrix protein 1, osteopontin, integrin alpha 6, matrix metalloproteinase 12, and integrin beta 5. While CX3CR1 is typically restricted to placental endothelial cells, preeclampsia is characterized by its aberrant expression on syncytiotrophoblasts alongside reduced endothelial CX3CR1, confirming dysfunctional angiogenesis as a central pathogenic mechanism [140,141]. CX3CL1-induced angiogenesis occurs through the activation of the Raf1/MEK/ERK kinase cascade and the PI3K/Akt/eNOS signaling pathway via CX3CR1 G-protein. Regression modeling has demonstrated an inverse correlation between serum CX3CL1 levels and vascularization index in preeclamptic placentas, contrasting with the positive correlation observed in healthy pregnancies [141].

The atypical chemokine receptor (ACKR) family, comprising six members (ACKR1-6), modulates chemokine availability through scavenging, degradation, or transcytosis rather than direct chemotaxis [142,143,144]. ACKR2, expressed in non-hematopoietic cells including placental villous trophoblasts [145,146], shows significantly reduced mRNA and protein expression in early-onset preeclampsia compared to term deliveries. In vitro studies using trophoblast cell lines demonstrate that ACKR2 influences trophoblast proliferation and apoptosis, with hypoxia downregulating its expression. ACKR2 deficiency promotes cellular apoptosis and elevates CCL2 levels [147], while increased apoptotic trophoblasts in preeclampsia may enter maternal circulation, triggering systemic inflammation [148].

CCL2 levels are markedly elevated in both plasma and placental tissue of preeclamptic patients, correlating with disease severity and serving as a reliable predictive biomarker [149]. Mechanistically, low Nrf-2 activity (linked to ROS) and fetal cell-free DNA (cffDNA) from apoptotic trophoblasts amplify CCL2 expression through AIM2 DNA sensor activation. Elevated CCL2 drives the expansion of proinflammatory CD14+CD11c+CD163− monocytes that promote Fas-mediated extravillous trophoblast apoptosis, disrupting placental implantation while suppressing protective CD14+CD11c+CD163+ monocytes. Concurrent increases in CCL2 and CXCL8 recruit monocytes to vascular walls, inducing endothelial damage akin to atherosclerotic lesions [150]. Targeting the CCL2/CCR2 axis presents a promising therapeutic avenue for preeclampsia, though achieving cell-specificity to avoid disrupting beneficial monocyte functions remains a significant challenge.

CXCL9 and CXCL10 mediate diverse biological functions including T-cell chemotaxis, endothelial adhesion, and NK cell-mediated cytolysis, with minimal effects on neutrophils and variable monocyte/B-cell responses [151]. Their elevation in preeclampsia reflects excessive Th1-derived TNF-α and IFN-γ production, linking systemic intravascular inflammation to pregnancy pathology. These chemokines exhibit potent anti-angiogenic properties [152], with NF-κB activation via TLR3/4 further amplifying their expression as “NF-κB-responsive genes” [55]. This multi-faceted chemokine dysregulation underscores their integrative role in preeclampsia’s inflammatory, apoptotic, and angiogenic disturbances.

## 10. Conclusions and Future Perspectives

The execution of genetically programmed functions for most cell types is intrinsically linked to cellular migration. This navigation process presents numerous challenges, as hundreds of cells with different objectives migrate simultaneously and in close proximity in response to a limited number of signals. Cells traverse significant distances through diverse environments of varying composition and density, which are capable of rapid dynamic changes. Nevertheless, constant adaptation of signal availability to specific migratory events occurs consistently.

The considerable diversity of chemokines and their associated signaling systems represents a more complex phenomenon than simple ligand–receptor interactions. Over the past decade, accumulating evidence demonstrates that chemokine functions extend beyond immune cell recruitment and gradient establishment. Chemokines facilitate cellular migration to organs enabling functional specialization, while also transmitting signals to other cells to execute programmed functions within their microenvironment or to migrate from it.

Successful pregnancy progression culminating in the birth of a healthy child requires the participation and interaction of numerous factors, including proper trophoblast invasion, proliferation, and differentiation, successful decidualization, and balanced maternal immune tolerance toward fetal cells. During pregnancy, the formation of the feto-maternal interface involves the intensive movement of immunocompetent cells and tissue morphogenesis. These processes particularly depend on chemokines, which establish crucial communication networks between fetal-derived trophoblast cells and maternal-derived decidual cells. However, abnormal chemokine function, chemokine–receptor interactions, and target cell engagements lead to trophoblast dysfunction, angiogenesis impairment, and breakdown in maternal–fetal immune tolerance, resulting in severe pregnancy complications that can threaten both maternal and fetal survival. Investigating chemokine regulation and function during pregnancy may facilitate the development of clinical treatments for pregnancy pathologies.

Although each pregnancy stage features distinct immunological states accommodating fetal tolerance, the spectrum of chemokines involved in each phase and in pathological development indicates that chemokines enhance the migration of cells performing diverse placental functions.

The chemokine system emerges not merely as a component of the immune system, but as a master regulator of the complex cellular dialogues essential for a successful pregnancy. Throughout this review, we have delineated its pivotal roles in orchestrating decidualization, guiding trophoblast invasion and placental development, maintaining feto-maternal tolerance, and ultimately triggering parturition. The system’s remarkable complexity—achieved through receptor-ligand promiscuity, alternative splicing, decoy receptors, and heterocomplex formation—provides the precision and redundancy necessary to navigate the distinct immunological phases of gestation. However, this very complexity means that its dysregulation can precipitate severe complications, such as preeclampsia and preterm birth, underscoring its dual nature as both a fundamental physiological mechanism and a source of pathology.

The profound involvement of chemokines in pregnancy pathologies positions them as promising candidates for translational applications, yet this potential is tempered by significant challenges. The distinct chemokine signatures associated with specific pregnancy complications offer a compelling avenue for early diagnosis and prognostication. Elevated levels of CXCL10, CCL2, and CX3CL1 show strong association with preeclampsia and intra-amniotic inflammation. A shift in the CCL20/CCR6 axis and alterations in CXCL9 and CXCL10 are implicated in the dysregulation of T-cell subsets in preterm labor.

The future of biomarker discovery lies in validating these signatures in large, diverse clinical cohorts and developing multiplex panels that can accurately stratify risk long before clinical symptoms manifest. A major hurdle will be defining gestational age-specific reference ranges to distinguish pathological elevations from normal physiological fluctuations.

Targeting specific chemokine pathways offers a novel strategic approach for managing pregnancy disorders. Key candidate axes include: the CCL2/CCR2 axis for mitigating excessive monocyte recruitment in preeclampsia. The CXCL12/CXCR4/CXCR7 axis to modulate trophoblast invasion and placental angiogenesis. The CX3CL1/CX3CR1 axis to dampen detrimental leukocyte infiltration in preterm labor.

However, the therapeutic targeting of chemokines faces the formidable challenge of achieving cell- and context-specificity to avoid disrupting their vital physiological functions in immune surveillance and tissue remodeling. Strategies such as the use of biased ligands, localized delivery systems, or targeting downstream signaling nodes may provide pathways to this specificity.

Future research directions should be guided by the need to bridge critical knowledge gaps. How are precise chemokine gradients established and maintained in vivo throughout gestation? Advanced imaging and spatial transcriptomics are needed to map these networks in real-time. How do specific maternal factors (e.g., obesity, metabolic syndrome, or infection) reprogram the gestational chemokine network to increase the risk of complications? Beyond chemotaxis, what are the full spectra of non-classical functions for chemokines in processes like autophagy, cell differentiation, and metabolic adaptation at the maternal-fetal interface?

In conclusion, the chemokine system constitutes the central coordinating language of pregnancy, governing processes from implantation to parturition. Investigating molecular and intercellular interactions during placentation is critically important since disruptions in this process are associated with the development of preeclampsia and a high probability of pathological preterm birth. The regulation of homeostatic, inflammatory, and morphogenetic processes by chemokines may underlie the transition from normal pregnancy to pathological conditions.

Understanding the mechanisms underlying these processes through contemporary high-throughput technologies will not only deepen our fundamental knowledge of reproductive biology but will also facilitate the development of innovative strategies for diagnosing, predicting, and ultimately treating the most severe pregnancy complications.

The journey from mechanistic understanding to clinical translation is complex, but the rewards—ensuring safer pregnancies and healthier outcomes for mother and child—are paramount.

## Data Availability

Not applicable.

## Abbreviations

dNK—decidual natural killer cells; Treg—regulatory T cells; oxLDL—oxidized low-density lipoproteins; Mph—macrophages; M1, M2—M1- and M2-polarized macrophages; Nph—neutrophils; Mn—monocytes; ECM—extracellular matrix; EVT—Extravillous trophoblast.
